# Integrated Traditional Chinese Medicine Improves Functional Outcome in Acute Ischemic Stroke: From Clinic to Mechanism Exploration With Gut Microbiota

**DOI:** 10.3389/fcimb.2022.827129

**Published:** 2022-02-09

**Authors:** Qian Guo, Can Ni, Linjing Li, Mo Li, Xiaoqing Jiang, Li Gao, Huaiqiu Zhu, Juexian Song

**Affiliations:** ^1^ Department of Neurology, Xuanwu Hospital, Capital Medical University, Beijing, China; ^2^ State Key Laboratory for Turbulence and Complex Systems, Department of Biomedical Engineering, College of Future Technology and Center for Quantitative Biology, Peking University, Beijing, China; ^3^ Department of Biomedical Engineering, Georgia Institute of Technology and Emory University, Atlanta, GA, United States; ^4^ Department of Neurology, Beijing Chaoyang Integrative Medicine Emergency Medical Center, Beijing, China

**Keywords:** acute ischemic stroke, integrated traditional Chinese medicine, therapeutic efficacy, gut microbiota, enterotype

## Abstract

As a life-threatening disease, stroke is the leading cause of death and also induces adult disability worldwide. To investigate the efficacy of the integrated traditional Chinese medicine (ITCM) on the therapeutic effects of acute ischemic stroke (AIS) patients, we enrolled 26 patients in the ITCM [Tanhuo decoction (THD) + Western medicine (WM)] group and 23 in the WM group. Thirty healthy people were also included in the healthy control (HC) group. ITCM achieved better functional outcomes than WM, including significant reduction of the phlegm-heat syndrome and neurological impairment, and improvement of ability. These facts were observed in different pretreatment gut enterotypes. In this paper, we collected the stool samples of all participants and analyzed the 16S rRNA sequence data of the gut microbiota. We identified two enterotypes (Type-A and Type-B) of the gut microbial community in AIS samples before treatment. Compared to Type-B, Type-A was characterized by a high proportion of *Bacteroides*, relatively high diversity, and severe functional damage. In the ITCM treatment group, we observed better clinical efficacy and positive alterations in microbial diversity and beneficial bacterial abundance, and the effect of approaching healthy people’s gut microbiota, regardless of gut enterotypes identified in pretreatment. Furthermore, we detected several gut microbiota as potential therapeutic targets of ITCM treatment by analyzing the correlations between bacterial abundance alterations and functional outcomes, where *Dorea* with the strongest correlation was known to produce anti-inflammatory metabolite and negatively linked to trimethylamine-N-oxide (TMAO), a biomarker of AIS. This study analyzed clinical and gut microbial data and revealed the possibility of a broad application independent of the enterotypes, as well as the therapeutic targets of the ITCM in treating AIS patients with phlegm-heat syndrome.

## 1 Introduction

Stroke is the leading cause of death and long-term neurological dysfunction in the world. A recent study on the global burden of disease, injury, and risk factors shows that the global stroke mortality rate has dropped significantly, and the incidence rate has declined slowly. However, the incidence rate is still rising in China, with 331 to 360 of every 100,000 people being patients, ranking the first in the world. The overall Chinese male lifetime stroke risk is as high as 40% (GBD 2016 Stroke Collaborators, 2019). Acute ischemic stroke (AIS) is the most common type of stroke (60%–80%) (Benjamin et al., 2017). Large artery atherosclerosis (LAA) is the primary cause (40%). It occurs from the sudden interruption or severe reduction of blood flow in the cerebral blood supply arteries, which leads to hypoxia and glucose deficiency in the brain tissue and, further, the accumulation of metabolic waste. Energy-dependent neurons will be damaged as a consequence. The affected area of brain tissue comprises two main damage areas: the core area and the surrounding area (also called the ischemic penumbra). During acute cerebral infarction, irreversible cell damage occurs in the core brain tissue area. In contrast, the penumbra area is damaged but not completely dysfunctional, which might be rescued by prompt and effective treatment (Lo, 2008). Thus, saving the ischemic penumbra is the primary task in the treatment of AIS. In the past few decades, several methods have been developed as the standard treatment. Thrombolytic technology and interventional therapy play an irreplaceable role, successfully saving the lives of a large number of patients and acquiring a satisfactory prognosis of stroke. However, the strict timeliness and high cost limited the promotion. In addition, conventional Western medicine (WM) for antiplatelet aggregation, lowering lipids, and improving circulation usually lead to complications, and some patients have poor compliance.

AIS belongs to the category of “stroke/apoplexy” in Chinese medicine. The pathogenesis of stroke is complicated. According to the theory of traditional Chinese medicine, it is generally caused by an imbalance of yin and yang and disturbance of circulation of qi and blood. Wind, fire, phlegm, and blood stasis are the main pathological factors to cause AIS. Traditional Chinese medicine (TCM) and WM have their own advantages and disadvantages in the treatment of stroke. With the development of the TCM diagnosis strategy, the combination model of WM diagnoses and TCM syndrome differentiation has significantly affected the treatment of acute stroke (Wang et al., 2020). The evolution of syndromes in the acute phase of stroke is complex, and the phlegm-heat syndrome is the primary syndrome in the acute phase of stroke (Wang and Xie, 2013). Additionally, the team of integrated Chinese and Western medicine in the Department of Neurology of Xuanwu Hospital has simplified the classification based on years of clinical experiences, dividing the syndrome into four types: phlegm-heat syndrome, phlegm-dampness syndrome, Yin deficiency syndrome, and Qi deficiency syndrome (Song et al., 2019a). Based on this, the treatment targeting the particular syndrome achieved good outcomes. Phlegm-heat syndrome is the most common among the four types. Clinically, in addition to the symptoms and signs of neurological deficits, AIS patients with the phlegm-heat syndrome also show bad breath, yellow tongue coating, red tongue, sticky sputum, thirst for cold drinks, dry stool, and yellow urine. In order to solve phlegm-heat syndrome, we created a Tanhuo decoction (THD) with the effects of clearing heat, removing phlegm, and eliminating turbid. The decoction is combined with WM treatment to improve both the TCM syndromes and neurological deficits (Guo et al., 2021). We also found that THD has the effects of anti-inflammation, antiplatelet aggregation, and reducing fibrinogen (Song et al., 2019b).

The gut microbiota plays an important and mysterious role in the communication between the brain and the gut. Many studies have confirmed that gut microbiota is related to the risk factors of ischemic stroke, such as obesity, hyperlipidemia, diabetes, and hypertension (Hold, 2014). Gut microbiota and microbial metabolites directly or indirectly affect the occurrence and development of stroke. Additionally, medications will have different efficacies in individuals with different microbial communities. Pretreatment gut enterotypes vary in the human population and are required to be considered in personalized medicine, especially the gut microbiota-associated intervention (Wang, 2013).

In this study, we enrolled 49 AIS patients with the phlegm-heat syndrome. The patients were randomly separated and treated with international (ITCM) (both THD and WM) (n = 26) and WM (n = 23) and HC (n = 30), respectively. We performed 16S rRNA gene sequencing of stool samples before and after treatments. We then evaluated the improvement in AIS patients’ neurologic impairment, daily life disability, and phlegm-heat syndrome and the microbial alterations after treatment. The increase of several beneficial bacteria after ITCM treatment had been reported in our previous study. In the current research, we first evaluated the therapeutic effects of ITCM in phlegm-heat syndrome AIS patients with different pretreatment gut enterotypes and explored therapeutic targets in the ITCM treatment. This study first revealed the broad application of ITCM in AIS with varying enterotypes under the challenge of personalized medicine.

## 2 Materials and Methods

We enrolled 49 AIS patients and 30 healthy controls in this study. All cases were AIS with phlegm-heat syndrome, hospitalized in the Department of Neurology, Xuanwu Hospital, Capital Medical University, from January 2017 to December 2018. We adopted two treatments, the ITCM (both THD and WM) treatment and the WM. All patients were randomly assigned into two treatment groups, 26 cases in the ITCM treatment group and 23 cases in the WM treatment group.

### 2.1 Inclusion Criteria and Exclusion Criteria

#### 2.1.1 Inclusion Criteria of AIS Patients in Treatment Groups

1. All selected cases met the diagnostic criteria of ischemic cerebrovascular disease at the 4th National Cerebrovascular Disease Conference and were confirmed by CT or MRI examination of the head within 3 days of onset.
2. The mechanism of infarction of the selected cases was classified into LAA type, according to TOAST (Trial of Org 10172 Acute Stroke Treatment) classification.
3. The duration from stroke onset to treatment was less than 1 week, and the clinical data were complete.
4. All the cases exhibited phlegm-heat syndrome in TCM syndrome differentiation.
5. Carotid artery ultrasound and transcranial color Doppler (TCCD) examinations were performed.
6. Vital signs are stable, and heart, liver, and kidney functions were normal.
7. All selected participants signed an informed consent form before the start of the study and were approved by the Ethics Committee of Xuanwu Hospital of Capital Medical University.

#### 2.1.2 Exclusion Criteria of AIS Patients in Treatment Groups

1. Non-ischemic diseases diagnosed as intracranial hemorrhage or other causes based on CT or MRI; hemorrhagic transformation;
2. Clear indications for anticoagulant therapy (cardiogenic embolism, such as atrial fibrillation and myocarditis);
3. Received intravenous thrombolysis or interventional treatment after the onset;
4. Patients with contraindications to aspirin;
5. Use antibiotics after coinfection;
6. Patients with arrangement for revascularization (interventional surgery or vascular surgery) within 3 months;
7. Pregnant women, breastfeeding women, or women of childbearing age who have a pregnancy plan within 3 months;
8. Severe heart, liver, and kidney dysfunction;
9. Incomplete clinical data record;
10. Patients who had been participating in clinical research of other drugs and equipment.

#### 2.1.3 Inclusion Criteria of Healthy Controls

1. There is no basic disease in any system of the body, and no organic lesions have been found in the recent physical examination.
2. Gender, age, height, weight, and other basic data match the treatment group.
3. Carotid ultrasound examinations were performed.
4. All selected subjects signed an informed consent form before the start of the study and were approved by the Ethics Committee of Xuanwu Hospital of Capital Medical University.

### 2.2 Determination of Risk Factors

Positive smoking history: smoking with at least one cigarette per day for more than 1 year or quitting smoking in less than 6 months. Positive drinking history: daily drinking >2 standard volumes (1 standard drinking volume is equivalent to 120 ml of wine, 360 ml of beer, or 45 ml of white wine). Blood pressure ≥ 140/90 mm Hg is hypertension. Diabetes symptoms: fasting blood glucose ≥ 7.0 mmol/l, or blood glucose in oral glucose tolerance test (2 h) ≥ 11.1 mmol/l, or random blood glucose ≥ 11.1 mmol/l. Dyslipidemia: total fasting plasma cholesterol in (TCH) ≥ 5.50 mmol/l, or triglyceride (TG) ≥ 1.69 mmol/l, or low-density lipoprotein cholesterol (LDL-C) ≥ 3.10 mmol/l, or high-density lipid protein cholesterol (HDL-C) < 0.90 mmol/l, or meeting multiple criteria above. Hyperhomocysteinemia: homocysteine ≥ 15 μmol/l. Obesity: body mass index (BMI) ≥ 28.0 kg/m^2^. All the criteria of the risk factors above are from the World Health Organization standards.

### 2.3 Carotid Ultrasound and Transcranial Color-Coded Duplex Sonography Inspection

The Philips IU22 color Doppler ultrasound diagnostic instrument from the Netherlands was used, and the 3.0–9.0-MHz linear array and 2.0–5.0-MHz convex array probes were selected. The inspection was performed by the attending physician and above in the ultrasound department. The common carotid artery, carotid artery bifurcated internal carotid artery, and external carotid artery were detected. The intima-media thickness (IMT), lumen inner diameter, and blood flow velocity were measured. The IMT value of ICA was taken as the measured value of the intima media of the posterior wall of the blood vessel at a distance of 1.0 cm from the carotid bulb distal to the CCA. According to the standards of Li et al. and Touboul et al. (Li and Hua, 2009; Touboul et al., 2012), IMT < 1.0 mm is normal; IMT ≥ 1.0 mm is intima-media thickness; and IMT > 1.5 mm or greater than the surrounding normal IMT value of at least 0.5 mm or more than 50% of the surrounding normal IMT value, and the local structural change protruding toward the lumen is plaque formation. According to the peak systolic flow rate (PSV), end-diastolic flow rate (EDV), and average flow rate (Vm) to determine the degree of stenosis. Mild stenosis: PSV < 155 cm/s, EDV < 60 cm/s; moderate stenosis: 155 ≥ PSV ≤ 230 cm/s, 60 ≥ EDV ≤ 100 cm/s; severe stenosis: PSV > 230 cm/s, EDV > 100 cm/s; occlusion: no blood flow signal.

In transcranial color-coded duplex sonography (TCCD) inspection, we chose a 1.6-MHz pulse-Doppler probe to routinely detect the main intracranial artery. The middle cerebral artery (MCA), anterior cerebral artery (ACA) A1 segment, and posterior cerebral artery (PCA) were detected through the temporal window; the vertebral artery (VA) and basilar artery (BA) were detected through the occipital window. We detected the hemodynamic parameters of each artery. Vm was used to determine the degree of intracranial artery stenosis. Mild stenosis: 90 cm/s ≥ Vm ≤ 120 cm/s; moderate stenosis: 120 cm/s ≥ Vm ≤ 150 cm/s; severe stenosis: 150 cm/s ≥ Vm; occlusion: no continuous blood flow signal was detected at the depth of 45–60 mm along the trunk of the middle cerebral artery.

### 2.4 Observation Indicators

We recorded the clinical information for all participants, including age, gender, BMI, smoking history, drinking history, diabetes history, hypertension history, hyperlipidemia history, carotid ultrasound, cerebral artery ultrasound, head CT, total cholesterol (TCH), high-density lipoprotein (HDL), low-density lipoprotein (LDL), triglyceride (TG), fasting blood glucose (GLU), C-reactive protein (CRP), fibrinogen (Fib), D-dimer, folic acid, vitamin B12, and blood homocysteine amino acid. Additionally, the National Institute of Health Stroke Scale (NIHSS) score, modified Rankin Scale (mRS) score, Barthel Index (BI) score, and fire-heat score were used to evaluate the neurologic impairment, recovery of prognosis, ability of daily life, and phlegm-heat syndrome in AIS, respectively.

### 2.5 Rating Scale

NIHSS < 4 is mild impairment in the scaling of neurologic impairment, while NIHSS ≥ 4 is moderate to severe (Kim et al., 2013).

0 ≤ mRS ≤ 2 represents a good prognosis in the scaling of prognosis, while 3 ≤mRS ≤ 6 stands for a poor prognosis (Duan et al., 2011).

mRS > 2 or BI < 85 is regarded as disabled in the scaling of daily life activity (Duan et al., 2011).

### 2.6 Scale of Fire-Heat Score

Phlegm-heat syndrome is mainly manifested as thick yellow moss, less saliva, red tongue, dry mouth, bitter mouth, sticky mouth, wanting cold drinks, breath, fear of heat, and dry stool with a strong smell. According to the theory of Chinese medicine, “heat is the gradual progress of the fire, fire is the pole of heat,” and “fire” and “heat” are the symptomatic features that describe the different stages of thermal imaging. According to the “Diagnostic Criteria for Stroke Syndrome” (1994) generated by the National Collaborative Group on TCM Encephalopathy Research of the State Administration of TCM, the syndrome elements such as tongue quality, tongue fur, pulse, expression, complexion, tone, taste, cold and heat, and the situation of urine and stool are mainly observed. Among them, tongue quality, tongue fur, and stool situation are the key elements of observation. In the scaling of the phlegm-heat syndrome, fire-heat score ≥ 7 is the lowest cutoff of the phlegm-heat syndrome; 7 ≤fire-heat score ≤ 14 represents mild syndrome; 15 ≤fire-heat score ≤ 22 represents moderate syndrome; and fire-heat score ≥ 23 represents severe syndrome. The rating scale is shown in **Supplementary Table 1**.

### 2.7 Diagnostic Criteria for Ischemic Stroke

1. Often onset in a quiet state;
2. No obvious headache and vomiting at the time of onset;
3. The onset is slow, and it gradually progresses or progresses in stages, which is related to cerebral atherosclerosis;
4. Clear awareness or mild disorder within 1–2 days after the onset;
5. There are symptoms and signs of the internal carotid artery system and/or vertebral–basal artery system;
6. CT or MRI results.

### 2.8 Treatment Groups

#### 2.8.1 Western Medicine Treatment Group

The Western medicine treatment includes the routine use of Western medicine and cerebral circulation. Aspirin enteric-coated tablets or clopidogrel is for antiplatelet aggregation. Atorvastatin calcium is used to lower lipid and stable plaque. Edaravone, alprostadil, and cinepazide maleate are used to improve circulation. The course of treatment is 7–10 days. Clinical data were recorded before and after treatment, including AIS indexes (fire-heat score, BI score, NIHSS score, and mRs score) and laboratory test results.

#### 2.8.2 Integrated Chinese and Western Medicine Treatment Group

Each unit of THD consists of 9 g Coptidis Rhizoma, 5 g Rhei Radix et Rhizoma, 9 g Forsythia, 9 g Lophatherum gracile, and 9 g Bile Arisaema. We purchased the conformed herbs, which met the standards of China Pharmacopoeia (2015 edition), from Sinopharm Group Beijing Huamiao Pharmaceutical Co., Ltd. The company performed quality control of the herbs and determined the main chemicals *via* high-performance liquid chromatography. The decoction was prepared by Xuanwu Hospital Capital Medical University according to the standard production process (Guo et al., 2021). Patients in the ITCM group orally took one dose decoction a day, a half for the morning and a half for the evening, for 7 days. Clinical data were recorded before and after treatment. The same group of trained neurologists evaluated the functional impairment of WM and ITCM patients.

### 2.9 Stool Sample Collection, DNA Extraction, and 16S rRNA Sequencing

We performed 16S rRNA gene sequencing of stool samples from 49 AIS patients before and after treatments and 30 healthy controls. Following the manufacturer’s instructions, the microbial community genomic DNA from the fresh stool samples was extracted using the E.Z.N.A.^®^ Soil DNA Kit (Omega Bio-tek, Norcross, GA, USA). The concentration and purity of DNA were monitored on 1% agarose gels. The primer pairs 338F (5′-ACTCCTACGGGAGGCAGCAG-3′) and 806R (5′-GGACTACHVGGGTWTCTAAT-3′) were selected to amplify the V3–V4 regions of the 16S rRNA gene in the extracted DNA. The PCR amplification procedure of 16S rRNA was set as follows: 95°C for 3 min (initial denaturation); 27 cycles of 95°C for 30 s (of denaturing), 55°C for 30 s (annealing) and 72°C for 45 s (extension); 72°C for 10 min (extension); stored at 4°C. Then, the PCR production was paired-end sequenced on an Illumina MiSeq PE300 platform (Illumina, San Diego, USA).

### 2.10 16S rRNA Analysis

We processed the demultiplexed paired-end sequences and constructed an amplicon sequence variant (ASV) table using the DADA2 pipeline in Qiime2 (version 2020.8.0) (Bolyen et al., 2019). The parameter settings were as follows: –p-trunc-len-f 295, –p-trunc-len-r 295, and default values for other parameters. We then annotated the ASVs using a naive Bayesian classifier method with 99% identity Greengenes rRNA database (version 13.8.99) (DeSantis et al., 2006). After that, the abundance matrices at the levels of phylum, class, order, family, and genus were created for each sample (Wang et al., 2019; Xu et al., 2020; Guo et al., 2021). The composition of gut microbiota of patients was typed into different enterotypes using the “DMM” R package (version 1.32.0) (Holmes et al., 2012). We chose the optimal number of enterotypes that minimized Laplace criteria. We performed principal coordinated analysis (PCoA) based on Bray–Curtis distance metrics to illustrate the taxonomic compositions of each sample using the *pcoa* function in R package “ape” (version 5.4.1) (Paradis and Schliep, 2019). Spearman correlation (R) between the bacterial abundance change and the clinical outcome was calculated using the *corr.test* function in “psych” R package (version 2.0.9) (Revelle, 2021), and the absolute correlation ≥ 0.35 was used as the cutoff. All the figures were plotted using R (version 4.0.2).

### 2.11 Statistical Analysis

Continuous variables were represented by mean ± standard deviation (SD). The two-tailed Student’s t-test with Welch’s correction and the two-tailed Wilcoxon rank-sum test were used to identify the differential continuous variables normally distributed and non-normally distributed, respectively. Differential discrete variables were tested using the chi-square test or two-tailed Fisher’s exact test. *p* ≤ 0.05 represented statistically significant.

## 3 Results

### 3.1 ITCM Achieved Better Clinical Outcomes Than WM

The patients enrolled in this study were all incipient AIS patients. There were no significant differences between the ITCM and WM groups in the basic information, especially in the risk factors of AIS, including age, BMI, blood cholesterol levels (TCH, LDL, HDL), smoking, hypertension, diabetes, and bad habits (drinking and smoking) (*p* > 0.05) (**Table 1**).

**Table 1:** The basic information of the ITCM treatment group and WM treatment group.

	ITCM group (n = 27)	WM group (n = 23)	*p*
Male, n (%)	15 (55.6%)	15 (65.2%)	0.569
Age, year	62.11 ± 15.32	56.22 ± 8.73	0.102
BMI, kg/m^2^	25.31 + 3.28	25.72 ± 4.44	0.953
Smoke, n (%)	12	10	1
Drinking, n (%)	9	7	0.303
Hypertension, n (%)	18 (66.7%)	17 (73.9%)	0.758
Diabetes, n (%)	7 (25.9%)	8 (34.9%)	0.548
Hyperlipidemia, n (%)	5 (18.5%)	4 (17.4%)	1
Stomach, n (%)	6 (22.2%)	4 (17.4%)	0.736
Folic acid, ng/mL	7.25 ± 2.57	9.869 ± 5.355	0.097
VitB12, pg/mL	375.63 ± 294.52	413.667 ± 253.991	0.383
HCY, μmol/L	15.98 ± 10.29	16.3 ± 9.926	0.778
HbA1C, %	5.85 ± 1.17	6.41 ± 1.93	0.572
GLU, mmol/L	5.86 ± 1.2	6.37 ± 2.37	0.984
TG, mmol/L	1.57 ± 0.67	1.66 ± 0.87	0.8
TCH, mmol/L	4.02 ± 0.87	4.12 ± 0.8	0.69
HD, mmol/L	1.06 ± 0.19	1.06 ± 0.23	0.778
LDL, mmol/L	2.47 ± 0.9	2.51 ± 0.68	0.884
CRP, mg/l	8.55 ± 7.98	17.461 ± 46.374	0.085
Cr, μmol/L	68.74 ± 19.79	62.74 ± 16.11	0.35
BUN, mmol/L	296.26 ± 94.5	302.78 ± 65.99	0.748
UA, μmol/L	4.82 ± 1.58	4.38 ± 1.26	0.34
AST, IU/L	23.19 ± 6.87	23.91 ± 9.77	0.853
ALT, IU/L	23.41 ± 17.53	23.22 ± 16.31	0.552
Fib, g/l	3.62 ± 0.98	3.57 ± 1.505	0.567
D-Dimer, μg/ml	0.75 ± 0.94	1.161 ± 2.366	0.651
TBA, μmol/L	4.45 ± 3.76	3.73 ± 3.41	0.34

The clinical outcomes were evaluated by the alterations of AIS indexes, including fire-heat, NIHSS, mRS, and BI scores. For fire-heat and NIHSS scores, we compared the values before and after treatments and compared the reduction of the values between treatment groups. The reduction of the two scores represented the alleviation of the phlegm-heat syndrome and neurological impairment, respectively. Based on mRS and BI scores, the numbers of disabled and abled patients before and after treatments were compared (abled, mRS ≤2 or BI ≥85).

The neurological impairment and phlegm-heat syndrome, reflected by NIHSS and fire-heat scores, were significantly reduced after either ITCM or WM treatments; however, disabled patients, reflected by mRS and BI scores, were only remarkably decreased in ITCM groups (*p* < 0.05, **Table 2**). Additionally, ITCM led to more reduction in NIHSS and fire-heat scores than WM (reduction of NIHSS, 2.11 ± 1.76 *vs.* 0.78 ± 0.85, *p* = 0.001; reduction of fire-heat score, 9.7 ± 2.35 *vs.* 3.13 ± 1.89, *p* = 0). These results revealed that ITCM achieved better clinical outcomes than WM.

**Table 2:** Comparison in ITCM group for pretreatment and posttreatment.

Treatment		Pretreatment	Posttreatment	Reduction	*p*
ITCM	NIHSS	4.33 ± 2.77	2.22 ± 2.03	2.11 ± 1.76	0.000***
	Fire-heat score	18.59 ± 5.06	8.89 ± 4.01	9.7 ± 2.35	0.000***
	mRS ≤ 2, n (%)	5	22	–	0.000***
	BI ≥ 85, n (%)	3	13	–	0.006**
WM	NIHSS	2.7 ± 2.48	1.91 ± 2.21	0.78 ± 0.85	0.001***
	Fire-heat score	16.91 ± 6.13	13.78 ± 5.71	3.13 ± 1.89	0.000***
	mRS ≤ 2, n (%)	13	18	–	0.208
	BI ≥ 85, n (%)	6	12	–	0.13

**p ≤ 0.01, ***p ≤ 0.001.

### 3.2 Characterization of Enterotypes in AIS Patients Before Treatment

**Figure 1:**
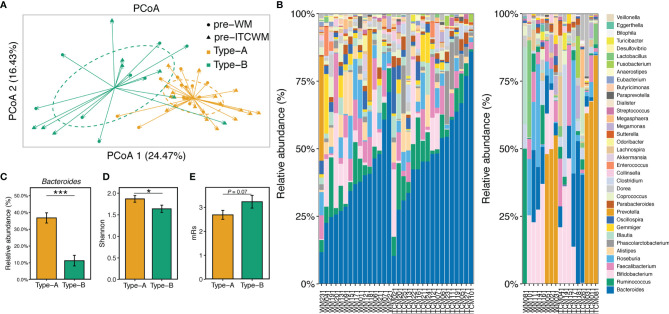
Characterization of gut enterotypes in AIS samples. **(A)** PCoA based on Bray-Curtis distances at ASV level showed different taxonomic compositions between the two enterotypes. **(B)** Microbiota communities on the genus level. The samples in the left and right bar plots were ordered by the abundance of *Bacteroides* and *Prevotella*, respectively. **(C)** The abundance of Bacteroides in the two enterotypes. **(D)** Microbial diversity (Shannon index) of the two enterotypes. **(E)** mRs scores of the two enterotypes. The colors of bars represented the enterotypes. Red, Wm treatment; Blue, ITCM treatment. *p ≤ 0.05, ***p ≤ 0.001.

Compared to HCs, 17 genera were differentially abundant in AIS samples before treatment, especially *Prevotella*, *Roseburia*, and *Ruminococcus* (**Supplementary Figure 1**). Using the *DMM* R package (version 1.32.0), the gut microbiota of all patients before treatment was divided into two distinct gut enterotypes designated as Type-A (containing 31 samples) and Type-B (containing 18 samples). There was no significant difference in the distribution of the two enterotypes between the two treatment groups (*p* > 0.05). The detailed analysis methods were included in *Materials and Methods*. PCoA analysis showed high similarity among samples in the same enterotype and apparent dissimilarities between different enterotypes (**Figure 1A**). The taxonomic composition of the 49 AIS samples (**Figure 1B**) further exhibited the detailed differences among enterotypes. Compared to Type-B, microbial communities of Type-A were dominated by a high level of *Bacteroides* (**Figures 1B, C**) accompanied by a higher alpha diversity (**Figure 1D**). These results revealed the non-negligible heterogeneity in the gut microbial composition of AIS patients.

There were no significant differences between the two enterotypes in the measurements of AIS risk factors (*p* > 0.05, **Supplementary Table 2**). However, AIS patients with Type-B microbiota exhibited more severe disability, reflected by higher mRs scores (*p* = 0.071) (**Figure 1E**). 

### 3.3 Better Achievements of ITCM in AIS Regardless of Gut Enterotypes

**Figure 2:**
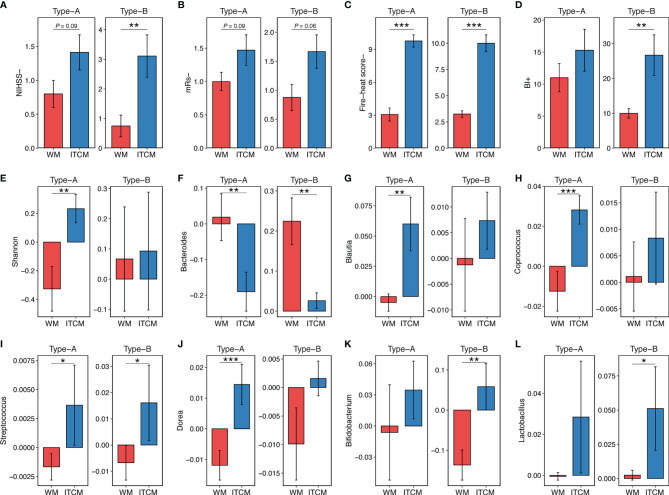
The outcomes of AIS patients with different gut enterotypes and different treatments. **(A–D)** Clinical outcome of AIS patients with different gut enterotypes and different treatments. **(E)** Increase of microbial diversity (Shannon index) of AIS samples with different gut enterotypes and different treatments. **(F–L)** Increase of genus abundance in AIS samples with different gut enterotypes and different treatments. The colors of bars represented the treatment types. Red, Wm treatment; blue, ITCM treatment. NIHSS-, the decrease of NIHSS score; mRs-, the decrease of mRs score; BI+, the increase of BI score; fire-heat score-, the decrease of fire-heat score. *p ≤ 0.05, **p ≤ 0.01, ***p ≤ 0.001.

ITCM achieved better therapeutic effects on both gut enterotypes, reflected by the significant reduction of NIHSS scores, mRs scores, fire-heat scores, and the remarkable increase of BI scores (**Figures 2A–D**). Also, the effect of ITCM was not weakened by the more severe pretreatment functional damage in Type-B patients. Instead, it led to more reduction of NIHSS scores in patients with Type-B gut enterotypes than Type-A (3.111 ± 2.147 *vs.* 1.412 ± 1.064, *p* < 0.05).

Different treatments resulted in different changes in the gut microbiota of patients with the same enterotype. ITCM showed superior improvement of microbial diversity, elevating the diversity in both enterotypes. By contrast, WM slightly increased the diversity in the Type-B enterotype but decreased it in the Type-A enterotype (**Figure 2E**). Additionally, ITCM drove the gut microbial composition of AIS patients closer to HCs (**Supplementary Figure 2**).

For patients with the Type-A enterotype, 18 and 15 genera approached healthy subjects in ITCM and WM groups, respectively (**Supplementary Figure 3A**). Eight genera altered reversely after the two treatments. Particularly, while *Bacteroides* (an important lipopolysaccharide (LPS)-producing genus) increased after WM treatment, it decreased after ITCM treatment (**Figure 2F**). Besides, SCFA-producing bacteria *Blautia*, *Coprococcus*, *Streptococcus*, and *Dorea* decreased in the WM group but increased in the ITCM group (**Figures 2G–J**). These results revealed more positive bacterial alterations in the Type-A gut enterotype with the ITCM treatment, including the improved microbial diversity, the elevated SCFA-producing bacteria, and the inhibited LPS-producing bacteria.

For patients with the Type-B enterotype, 15 and 14 genera approached healthy subjects in the ITCM and WM groups, respectively (**Supplementary Figure 3B**). Two genera exhibited opposite changes in the two treatment groups. *Streptococcus* and *Bifidobacterium* (SCFA-producing bacteria) decreased in the WM group but increased in the ITCM group (**Figures 2I, K**). *Lactobacillus*, another SCFA-producing genus, also increased in the ITCM group (**Figure 2L**). In addition, *Bacteroides* were significantly increased after WM treatment but only slightly changed with ITCM treatment (**Figure 2F**). These results further disclosed ITCM’s advantages in the Type-B enterotype.

Our data proved the satisfactory effect of ITCM on clinical outcome, microbial diversity, and the elevation of beneficial genera, regardless of the enterotypes of AIS patients. By contrast, WM showed inferior therapeutic effects and exhibited unsatisfactory regulations of the microbial community in patients with different gut enterotypes.

### 3.4 The Functional Outcomes of AIS Were Closely Related to the Gut Microbiota

As mentioned above, the alleviation of phlegm-heat syndrome and neurological impairment was more significant in the ITCM group than that in the WM group (*p* < 0.05). By analyzing the correlation of the changes in the abundance of gut microbiota after treatments, we found that the superior therapeutic effect of ITCM may be related to the increase of some beneficial genera and the decrease of harmful genera in the gut microbiota of AIS patients.

**Figure 3:**
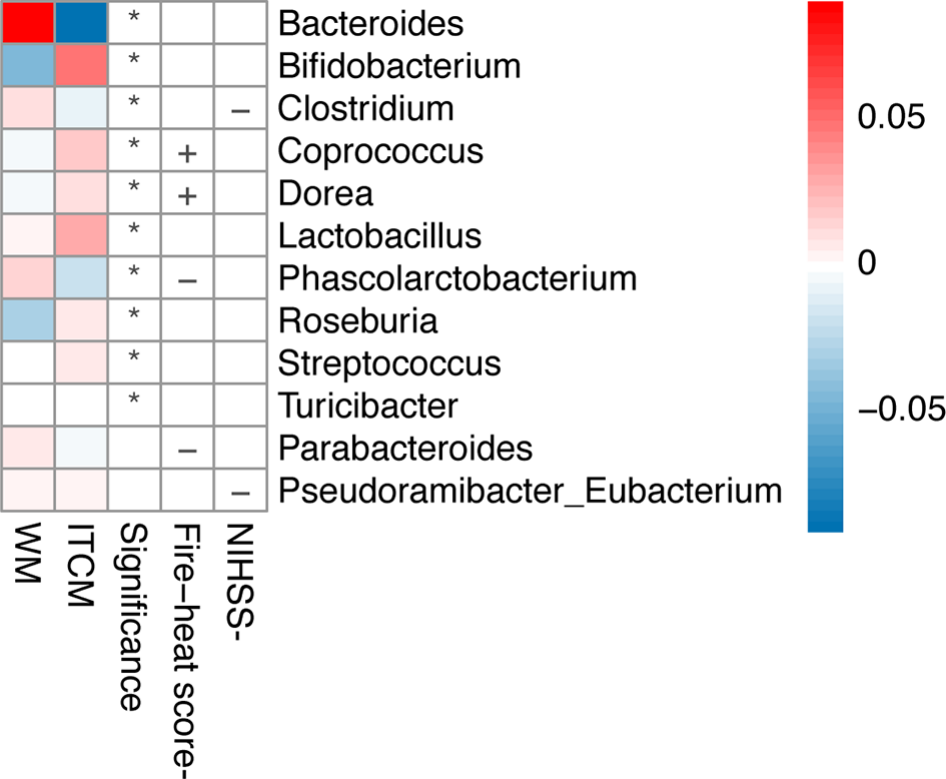
Genera changed differentially in the two treatment groups and correlations between the change of genus and clinical outcome. The first two columns represented the abundance changes of genera which were differentially altered in the two treatment groups. The third column represented the significance of abundance changes in the between-group comparison (Wilcoxon rank-sum test, *p* < 0.05). The last two columns represented the correlations between the change of genus and clinical outcome, where the clinical outcomes were evaluated by the decrease of NIHSS score or the fire-heat score *p ≤ 0.05; +, positive correlation; -, negative correlation.

The alleviation of phlegm-heat syndrome (reduction of fire-heat score) in AIS patients was positively correlated with *Coprococcus* and *Dorea* but negatively related to *Parabacteroides* and *Phascolarctobacterium*. The decrease of neurological impairment (reduction of NIHSS score) of AIS patients was negatively correlated with *Clostridium* and *Pseudoramibacter_Eubacterium* (**Figure 3**). We observed that all the bacteria positively correlated with functional outcomes were significantly upregulated after ITCM treatment but significantly downregulated after WM treatment; most of the bacteria negatively correlated with functional outcomes were significantly downregulated after ITCM treatment, but upregulated after WM treatment (**Figure 3**). In particular, in the ITCM group, *Coprococcus* and *Dorea* were significantly upregulated, while *Clostridium*, *Parabacteroides*, and *Phascolarctobacterium* were significantly downregulated. By contrast, reverse alterations were exhibited in the WM group.

## 4 Discussion

The pathogenesis of acute ischemic brain injury is complex, with many pathogenic factors. Modern pathological studies show that the occurrence of acute cerebral infarction is mainly connected with oxidative stress, inflammatory injury, immune injury, free radical injury, and other mechanisms (Manzanero et al., 2013). When cerebral ischemia occurs, the signal transduction system associated with the inflammatory response is activated in the human body, resulting in higher levels of inflammatory factors and mediators, such as C-reactive protein, interleukin, tumor necrosis factor, and coagulation factors (Acalovschi et al., 2003; Sánchez-Moreno et al., 2004). Elevated inflammatory levels result in the accumulation of large amounts of metabolites and then the severe irreversible damage of brain tissue, and consequently poor prognosis (Wang et al., 2014).

Understanding the etiology and pathogenesis of stroke is a continuous process improved by medical doctors of all generations in traditional Chinese medicine. From the “outer wind” treatment before Tang and Song dynasties to the “inner wind” theory of Jin and Yuan dynasties, then to the contemporary treatment of stroke targeting on “fire and poison” (Wang et al., 2015), “real heat” is always the therapeutic target (Meng and Gao, 2015). Theories of traditional Chinese medicine say: “The spleen and stomach are the foundation of life” and “stomach heat is the cause of blood turbidity”. The stomach heat, fluid transpiration, and thick blood facilitate the accumulation of metabolic waste and increase blood turbidity, slowing blood flow and aggravating blood stasis. Insufficiency of blood stasis generates internal heat, and then phlegm and blood stasis heat together form plaque. These cascading responses increase blood vessel stenosis and cerebrovascular accident. Based on the syndrome characteristics of cerebrovascular disease, Professor Gao Li combined modern etiology with pathology and imaging examination in syndrome analysis. The team of Gao also simplified the TCM syndromes of AIS into four types, where the phlegm-heat syndrome is the most common (Song et al., 2019a). In addition to neurological deficits, AIS with phlegm-heat syndrome exhibits bad breath, thick tongue coating, sticky sputum, abdominal distension, dry or poor stools, etc. According to these syndrome characteristics, we have developed THD to clear phlegm and heat. Rhei Radix et Rhizoma and Lophatherum Gracile can clear heat; Forsythia plays a role in detoxification; Coptidis Rhizoma has the function of removing dampness and heat; and Bile Arisaema clears heat and phlegm.

In this study, we used integrated medicine, including THD and WM, to treat AIS patients with phlegm-heat syndrome according to the principle of TCM syndrome differentiation, and set a WM treatment group. There was no statistical difference between the two treatment groups before treatment in terms of the measurements of AIS risk factors (*p* > 0.05). After treatment, we observed the significant clinical outcomes in the ITCM group in terms of better alleviation of the phlegm-heat syndrome and neurological impairment, and decreased numbers of disabled patients, compared to the WM group.

To illustrate the underlying mechanisms of the ITCM treatment at the microbial level, we analyzed the gut microbiota data before and after treatment. Our previous study observed a better improvement of several beneficial bacteria after ITCM treatment than after WM treatment (Guo et al., 2021). In the current study, we evaluated the effects of ITCM in different gut enterotypes. Due to the different taxonomic compositions, enterotypes exhibited distinguished metabolic patterns. Christensen et al. reported that different strategies were preferred in different enterotypes in a review on obesity management (Christensen et al., 2018). They pointed out enterotype as a pretreatment biomarker and emphasized its importance in the personalized management of patients. In our study, two gut enterotypes (Type-A and Type-B) were identified in the microbial communities of AIS samples before treatment. No dominant microbiota was identified in Type-B. By contrast, the Type-A enterotype was characterized by a high level of *Bacteroides*, higher diversity, and slighter functional damage of AIS. The heterogeneity caused by different enterotypes aroused our attention to the therapeutic effects of ITCM and WM treatments in different enterotypes. By comparing both clinical outcomes and microbial alterations with different treatments in a specific enterotype, we observed more robust effects of ITCM in all enterotypes. The advantages could be reflected by the superior clinical outcomes, the elevation of microbial diversity and beneficial genera, and the effect of approaching health people’s gut microbial composition. The clinical outcomes included the alleviation of the phlegm-heat syndrome, neurological impairment and disability, and the improvement of ability. Our data revealed the possibility of a broad application of ITCM treatment in AIS patients with the phlegm-heat syndrome.

The enterotypes identified in this study were confirmed by the previous studies to some extent (Arumugam et al., 2011; Liu et al., 2016). The *Bacteroides*-dominant Type-A enterotype was the most common enterotype in earlier researches. Another enterotype that was frequently reported was *Prevotella* dominated. Although this enterotype was not identified in the current study, some microbial samples in the Type-B enterotype (non-dominant microbiota) exhibited a high proportion of *Prevotella*. The developers of DMM methods (Holmes et al., 2012), which were applied for community typing in this study, mentioned that the number of community types was likely to grow with data size. Therefore, we believed that the *Prevotella*-dominated enterotypes could be identified in AIS patients with an enlarged sample size in the future. Moreover, more robust conclusions will be made at that time. Despite this pity, our data were still an innovative study that compared the ITCM and WM treatments in different enterotypes and evaluated the rationality of the universal promotion of ITCM in AIS patients. With the rising attention paid to the differential gut microbial composition in diagnosing and treating AIS, ITCM will increasingly exhibit its broad application value.

To investigate the mechanism of ITCM on the microbial level, we evaluated the correlations between the alterations of gut microbiota and the clinical outcome. We identified the bacteria which were altered, accompanied by a positive clinical outcome. The alleviation of the phlegm-heat syndrome and neurological impairment was positively correlated with *Coprococcus* and *Dorea* but negatively related to *Clostridium*, *Parabacteroides*, and *Phascolarctobacterium*. While *Coprococcus* and *Dorea* were upregulated after ITCM treatment, *Clostridium*, *Parabacteroides*, and *Phascolarctobacterium* decreased. The alterations were opposite in the WM group. In our previous study, according to previous studies and our findings, we proposed a hypothesis that ITCM will mediate the gut bacteria and subsequently downregulate the sterile inflammation and platelet aggregation by reducing the levels of LPS and TMAO and then inhibit the aggravation of AIS (Guo et al., 2021). In the current study, *Dorea*, *Coprococcus*, and other four bacteria (*Blautia*, *Streptococcus*, *Bifidobacterium*, *Lactobacillus*) were upregulated by ITCM in both Type-A and Type-B patients. Some of them have negative associations with LPS and TMAO (Riedel et al., 2006; Rodes et al., 2013; Gregory et al., 2015; Manor et al., 2018; Qiu et al., 2018; Borges et al., 2019). Berberine and Rhein, the active ingredients in Coptidis Rhizoma and Rhei Radix et Rhizoma, were reported to enhance these bacteria (Zhang et al., 2012; Zhang et al., 2015; Zhang et al., 2020). These pieces of evidence further supported our previous hypothesis and provided its reasonability in AIS patients with different enterotypes.

In this study, we found that ITCM was more effective in AIS patients with the phlegm-heat syndrome, regardless of the pretreatment gut enterotypes. It could not only improve the TCM symptom characteristics but also promote the recovery of neurological function and daily life ability. In our previous study, we also observed a lower incidence of cerebrovascular events within 3 and 6 months after the treatment in the ITCM group than in the WM group, which revealed that the long-lasting benefit after ITCM treatment is completed. The long-term benefit of ITCM on gut microbiota is an important topic that requires further exploration; nevertheless, our current study is strong evidence of the satisfactory effects of ITCM from clinic to gut microbiota.

## Data Availability Statement

The datasets presented in this study can be found in online repositories. The names of the repository/repositories and accession number(s) can be found as follows: https://www.ncbi.nlm.nih.gov/, PRJNA683157.

## Author Contributions

JS and HZ co-supervised and designed the study. JS, LL, and LG performed the clinical management and data acquisition. QG and CN conducted the data analysis and result arrangement. QG, CN, ML, and XJ helped with plots and tables for the analysis and sequencing data processing. JS, QG, and CN wrote the manuscript. HZ and JS reviewed and revised the manuscript. All authors contributed to the article and approved the submitted version.

## Funding

This work was supported by the National Key Research and Development Program of China (2021YFC2300300), the Science and Technology Program of Beijing (Z171100001717012), the Key Special Project of Ministry of Science and Technology Research on modernization of Traditional Chinese Medicine (2019YFC1712400), and the National Natural Science Foundation of China (32070667, 31671366).

## Conflict of Interest

The authors declare that the research was conducted in the absence of any commercial or financial relationships that could be construed as a potential conflict of interest.

## Publisher’s Note

All claims expressed in this article are solely those of the authors and do not necessarily represent those of their affiliated organizations, or those of the publisher, the editors and the reviewers. Any product that may be evaluated in this article, or claim that may be made by its manufacturer, is not guaranteed or endorsed by the publisher.
