# Supplementary material for: Integrated Traditional Chinese Medicine Improves Functional Outcome in Acute Ischemic Stroke: From Clinic to Mechanism Exploration With Gut Microbiota
Source: Front Cell Infect Microbiol. 2022 Feb 9;12:827129. doi: 10.3389/fcimb.2022.827129 (PMC8877419; doi:10.3389/fcimb.2022.827129)
Supplement: Supplementary file 1 [file DataSheet_1.zip › DataSheet/Supplementary_Table_2.docx]

**Supplemental Table 2.** The basic information of AIS patients with Type-A and Type-B pre-treatment gut enterotypes.

|  | Type-A (n = 31) | Type-B (n = 18) | P |
| --- | --- | --- | --- |
| Sex | 22 (71.0%) | 8 (44.4%) | 0.218 |
| Smoke | 17 (54.8%) | 5 (27.8%) | 0.29 |
| Drink | 12 (38.7%) | 4 (22.2%) | 0.578 |
| Anterior | 17 (54.8%) | 7 (38.9%) | 0.234 |
| Stroke | 8 (25.8%) | 5 (27.8%) | 0.746 |
| Diabetes | 10 (32.2%) | 5 (27.8%) | 1 |
| Hyperlipidemia | 5 (16.1%) | 4 (22.2%) | 0.7 |
| Stomache | 4 (12.9%) | 6 (33.3%) | 0.075 |
| Age | 56.78±12.13 | 63.41±13.5 | 0.159 |
| ALT | 23.41±15.98 | 24±18.89 | 0.916 |
| AST | 23.06±7.57 | 24.94±9.44 | 0.592 |
| BMI | 25.62±3.81 | 25±3.88 | 0.651 |
| BUN | 300.28±74.06 | 293.53±98.01 | 0.674 |
| Cr | 65.69±18.65 | 66.41±18.63 | 0.866 |
| CRP | 12.346±35.996 | 12.261±18.036 | 0.246 |
| D-Dimer | 1.07±2.06 | 0.613±0.734 | 0.562 |
| Fib | 3.56±1.4 | 3.661±0.891 | 0.353 |
| Folic acid | 8.403±4.138 | 8.59±4.477 | 0.633 |
| GLU | 5.91±1.82 | 6.47±1.9 | 0.186 |
| HbA1c | 5.96±1.58 | 6.42±1.6 | 0.226 |
| HCY | 15.5±8.26 | 16.9±13.243 | 0.734 |
| HDL | 1.07±0.2 | 1.04±0.21 | 0.777 |
| LDL | 2.58±0.74 | 2.29±0.9 | 0.172 |
| TBA | 3.79±3.16 | 4.75±4.4 | 0.528 |
| TCH | 4.14±0.82 | 3.88±0.85 | 0.144 |
| TG | 1.65±0.77 | 1.53±0.78 | 0.482 |
| UA | 4.89±1.59 | 4.04±0.93 | 0.133 |
| VitB12 | 351.581±189.963 | 486.562±385.163 | 0.466 |
