# Supplementary material for: Integrated Traditional Chinese Medicine Improves Functional Outcome in Acute Ischemic Stroke: From Clinic to Mechanism Exploration With Gut Microbiota
Source: Front Cell Infect Microbiol. 2022 Feb 9;12:827129. doi: 10.3389/fcimb.2022.827129 (PMC8877419; doi:10.3389/fcimb.2022.827129)
Supplement: Supplementary file 1 [file DataSheet_1.zip › DataSheet/Supplementary_Table_1.docx]

**Supplemental Table 1.** Scoring rules of phlegm-heat syndrome.

| Items | Symptoms | Scores |
| --- | --- | --- |
| Tongue | Red tongue | 5 |
|  | Deep red tongue | 6 |
| Tongue coating | Thin-yellow coating | 2 |
|  | Thick-yellow coating | 3 |
|  | Dry coating | 4 |
|  | Gray-black-dry coating | 5 |
| Excrement | Astriction within three days | 3 |
|  | Astriction more than three days | 4 |
| Expression | Irritability | 2 |
|  | Agitated and restless | 3 |
|  | Delirium | 4 |
| Face breathe odor | Loud, rough, or dry-red lips | 2 |
|  | Flame, panting, or halitosis | 3 |
| Fever | Fever | 3 |
| Pulse | Rough pulse | 2 |
| Mouth Feeling | Bitter taste and dry throat | 1 |
|  | Thirst with a predilection for cold drink | 2 |
| Urine | Odynuria | 1 |
