# Supplementary material for: Integrated Traditional Chinese Medicine Improves Functional Outcome in Acute Ischemic Stroke: From Clinic to Mechanism Exploration With Gut Microbiota
Source: Front Cell Infect Microbiol. 2022 Feb 9;12:827129. doi: 10.3389/fcimb.2022.827129 (PMC8877419; doi:10.3389/fcimb.2022.827129)
Supplement: Supplementary file 1 [file DataSheet_1.zip › DataSheet/Supplementary information legends.docx]

**Supplemental Figure**

**Supplemental Figure 1.** Differential genera between AIS patients and healthy controls.

* *p* ≤ 0.05, ** *p* ≤ 0.01, *** *p* ≤ 0.001.

**Supplemental Figure 2.** Increased Euclidean distance (IED) to gut microbiota of healthy controls.

(A) IED to healthy controls' gut microbiota for Type-A enterotype patients after WM treatment (left) or ITCM treatment (right). (B) IED to healthy controls' gut microbiota for Type-B enterotype patients after WM treatment (left) or ITCM treatment (right).

**Supplemental Figure 3.** Changes of the absolute difference between each bacterium of healthy controls and AIS patient after treatment.

(A) Changes of the absolute difference between each bacterium of healthy controls and AIS patients with Type-A enterotype after treatment. (B) Changes of the absolute difference between each bacterium of healthy controls and AIS patients with Type-B enterotype after treatment. Significance columns represent the statistical significance of the change values between WM and ITCM groups. Blue and red cells represent shrunk differences and enlarged differences, respectively. * *p* ≤ 0.05.

**Supplemental Table**

**Supplemental Table 1.** Scoring rules of phlegm-heat syndrome.

**Supplemental Table 2.** The basic information of AIS patients with Type-A and Type-B pre-treatment gut enterotypes.
