# Supplementary figures and images for: Integrated Traditional Chinese Medicine Improves Functional Outcome in Acute Ischemic Stroke: From Clinic to Mechanism Exploration With Gut Microbiota
Source: Front Cell Infect Microbiol. 2022 Feb 9;12:827129. doi: 10.3389/fcimb.2022.827129 (PMC8877419; doi:10.3389/fcimb.2022.827129)

**A**

Type-A

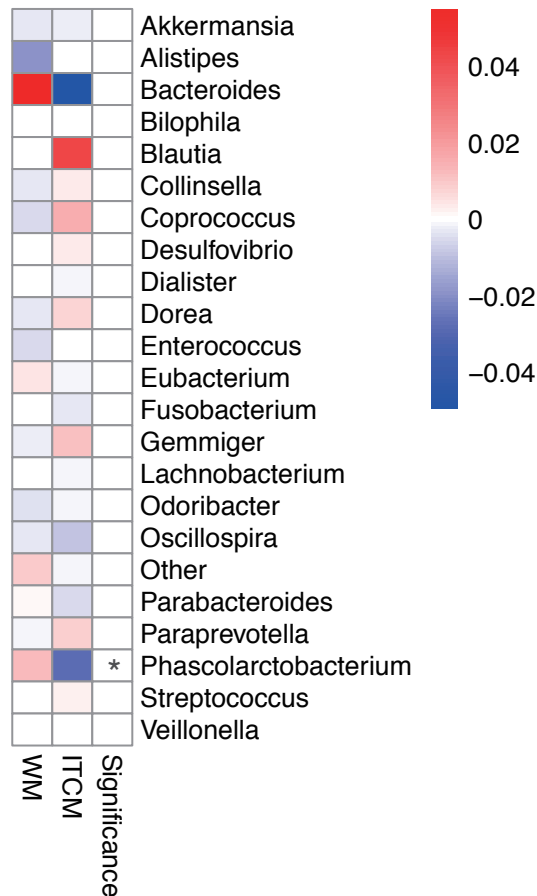**B**

Type-B

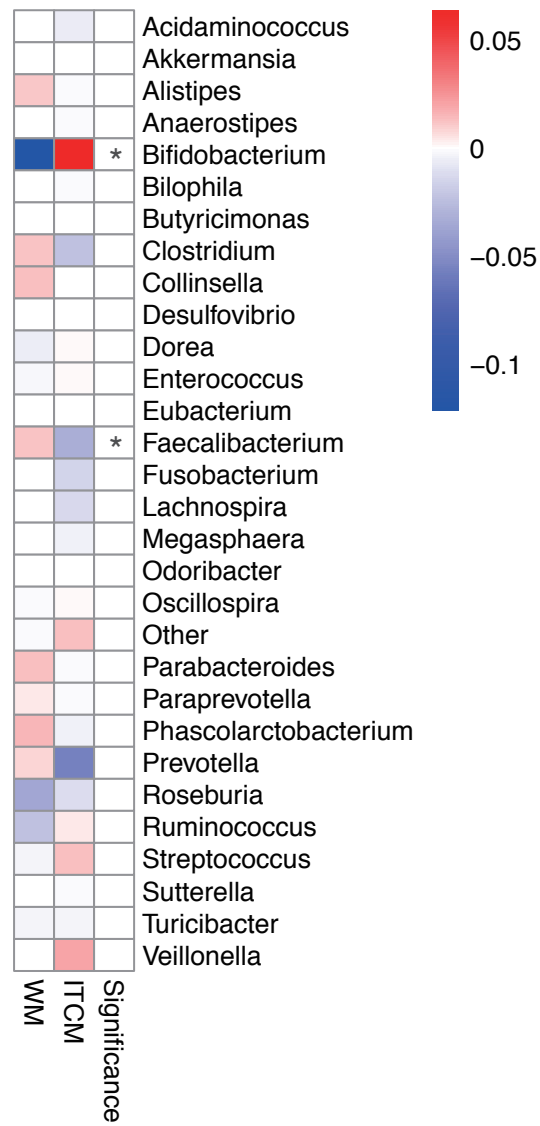

Supplement: Supplementary file 1 [file DataSheet_1.zip › DataSheet/Supplementary_Figure_3.pdf]

**A** Type-A

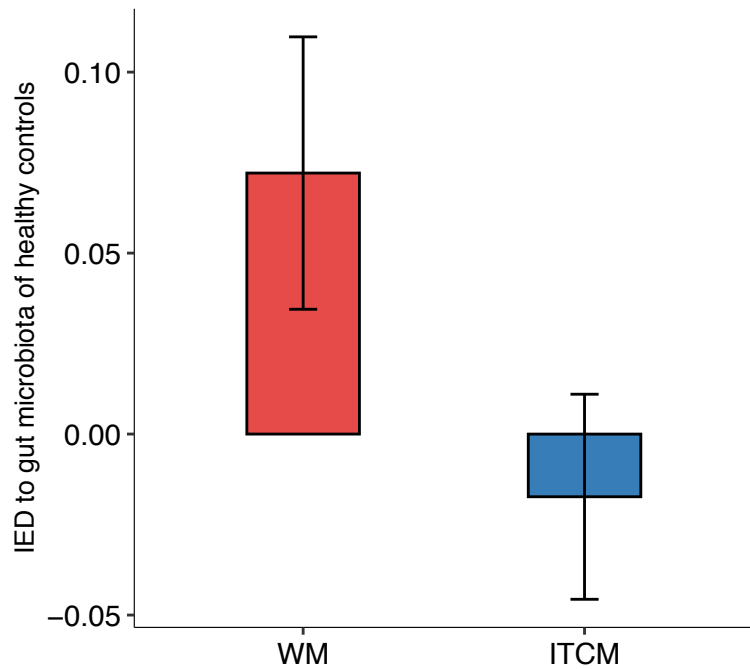

**B** Type-B

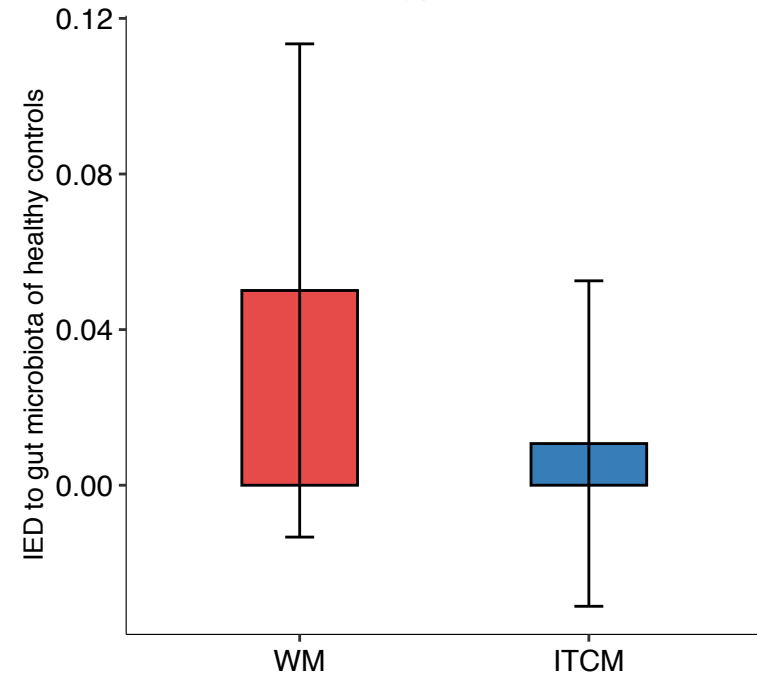

Supplement: Supplementary file 1 [file DataSheet_1.zip › DataSheet/Supplementary_Figure_2.pdf]

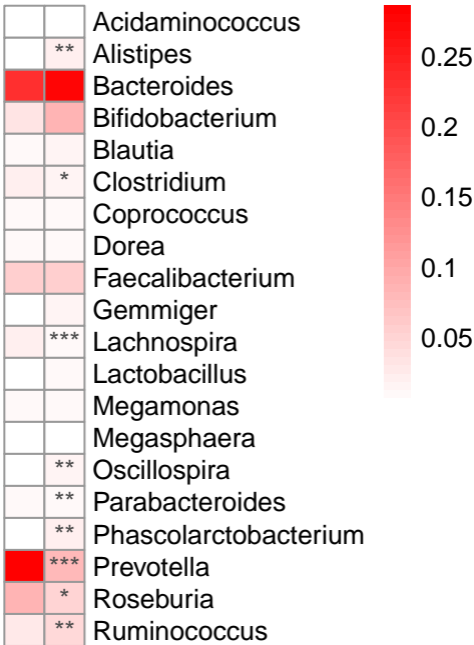

AIS  
Healthy-Group

Supplement: Supplementary file 1 [file DataSheet_1.zip › DataSheet/Supplementary_Figure_1.pdf]
